# BP Neural Network Could Help Improve Pre-miRNA Identification in Various Species

**DOI:** 10.1155/2016/9565689

**Published:** 2016-08-22

**Authors:** Limin Jiang, Jingjun Zhang, Ping Xuan, Quan Zou

**Affiliations:** ^1^School of Computer Science and Technology, Tianjin University, Tianjin 300350, China; ^2^School of Information and Electrical Engineering, Hebei University of Engineering, Handan 056038, China; ^3^School of Computer Science and Technology, Heilongjiang University, Harbin 150080, China; ^4^State Key Laboratory of Medicinal Chemical Biology, Nankai University, Tianjin 300074, China

## Abstract

MicroRNAs (miRNAs) are a set of short (21–24 nt) noncoding RNAs that play significant regulatory roles in cells. In the past few years, research on miRNA-related problems has become a hot field of bioinformatics because of miRNAs' essential biological function. miRNA-related bioinformatics analysis is beneficial in several aspects, including the functions of miRNAs and other genes, the regulatory network between miRNAs and their target mRNAs, and even biological evolution. Distinguishing miRNA precursors from other hairpin-like sequences is important and is an essential procedure in detecting novel microRNAs. In this study, we employed backpropagation (BP) neural network together with 98-dimensional novel features for microRNA precursor identification. Results show that the precision and recall of our method are 95.53% and 96.67%, respectively. Results further demonstrate that the total prediction accuracy of our method is nearly 13.17% greater than the state-of-the-art microRNA precursor prediction software tools.

## 1. Introduction

MicroRNAs are some of the most important noncoding RNA genes with rather short length. They regulate the expression of whole organism genes at the posttranscriptional level [1]. miRNA is widely involved in the metabolic activity of the body as well as in many important life processes, including cell proliferation and apoptosis, cell differentiation, growth and development of plants and animals, and organ formation [2–4]. Recently, several studies have shown that microRNAs are related to several cancers [5–7] and other diseases [8–10]. Caligiuri et al. [11] proposed that methods and compositions involving miRNAs are useful for the treatment of various diseases and cancers. Some miRNAs are regarded as potential therapeutic targets for various diseases [12]. Recently, the target gene (cancer gene) drugs, which developed in accordance with the theory on miRNA's gene silencing, have been used for incurable disease that has become a threat to human health problems for years [13]. In addition, the viral genome can encode a large number of miRNAs by itself. Through combination with target genes and coding by viruses or host cell, these miRNAs can lead to immune escape or antiviral effect against the host cell. Therefore, the accurate prediction of miRNA and its target genes, as well as the correct understanding of miRNA mechanism, has important practical significance in medical treatments. Thus, the research on novel miRNA identification is rather essential.

Feature selection mainly dominated the performance of the prediction model in the machine learning process [14–20]. In addition, effective features can represent the characteristics of the entire sequence data, which enables easy-to-build better prediction model. To represent the microRNA precursors, Xue et al. [21] proposed 32D novel triplet features, which involved secondary structure information. Jiang et al. [22] found that random rearrangement of the sequence could help obtain significant free-energy features. However, the free-energy computation for many random rearrangement sequences is very time consuming. Wei et al. [23] combined Xue et al.'s features and triplet nucleotide frequency to 98D features and obtained good performance result in human pre-miRNA identification. However, more features would not mean better performance because of some irrelevant and redundant features in the high dimensional or ultra-high dimensional feature set. The purpose of feature selection is to eliminate the irrelevant and redundant features of the feature set. In addition, the training time could be reduced effectively by the feature selection optimization [24]. Some studies focus on developing computational predictors by incorporating the sequence-order or structure-order effects [25, 26]. Several works indicated that proper features could improve the prediction performance of classification in a certain extent. For example, Wang et al. [27] employed the feature selection techniques to optimize the features in miR-SF. They proved that an optimized feature subset could improve the prediction performance. In addition, some popular recently proposed multiobjective optimization evolutionary algorithms can also be used as a possibly promising feature selection approach [28, 29].

Another factor that affects the performance of machine learning prediction method is the classifier algorithm. The selection of different classifiers often leads to the difference of classification results. Several different classifiers and strategies were employed for miRNA identification. Bayesian classifier algorithm was tested for predicting miRNA across different species in 2006 [30]. The method also utilized the multiple species of miRNA sequences and structural features. It proved that miRNA genes could be detected effectively in large scale of different species genomes.

MiPred classifier was tested for predicting miRNA in 2007 [22]. The method utilized random forest classifier algorithm. The prediction accuracy of MiPred is 10% higher than that of Triplet-SVM; the sensitivity and specificity of MiPred can reach to 95.09% and 98.21%. CSHMM classifier was also used for mining miRNA sequences from the genome [31], which utilized the Markov model. Overall, the accuracy of machine learning algorithm was up to 90%. The machine learning method is more accurate than the other methods.

In this study, we chose backpropagation neural network as the classifier. It has three advantages, including better generalization performance, faster learning speed, and good learning ability.

## 2. miRNA Identification with BP Neural Network

### 2.1. Pre-miRNA Features

#### 2.1.1. *n*-Gram Frequency

Some studies showed that the local primary sequence is crucial to the pre-miRNA sequence [32]. Thus, the *n*-gram frequency is often applied for the feature map in the selection of the primary sequence feature [33, 34]. However, no good methods are still available for tuning the value of *n*. In general, we choose *n* by comparing the effect of *n*-gram frequency with different *n*-values. In our feature set, we select the different *n* values (*n* = 2,3, 4) for comparison. The different frequency characteristics have almost the same effect on the classifier. Thus, consider that its base and adjacent base have practical biological significance. We chose *n* as 3. A total of 64 (64 = 4^3^)-dimensional frequency features were calculated.

#### 2.1.2. Triple Structure Sequence

In addition to high specificity of the primary sequence features, the secondary structure sequence of pre-miRNA is also a contributing factor. To analyze the contribution of the secondary structure, the secondary structure prediction software RNAfold is used to calculate the potential structures. In the secondary structure, each nucleotide of the sequence corresponds to two states, matching and nonmatching: record matching as “(” or “)” and nonmatching as “·.” In the structure, three character groups are considered as a unit, and every “)” is replaced as “(.” Thus, 8 (8 = 2^3^) different combinations are available as a unit, including “(((,” “((·,” “(·(,” “·((,” “(··,” “·(·,” “··(,” and “···.”

To characterize pre-miRNA sequence better, the first nucleotide of the corresponding subsequence was added to the front of each structure unit. This provides 32 (32 = 4 × 8) different combinations, that is, “A(((,” “U((·,” …, “G·((,” “G···.” For a sequence, the occurrence frequency of each combination is determined and coded into the 32D feature vector as the input of the classifier. This calculated 32D triple structure sequence feature is used to train the SVM classifier; the inclusion of the SVM classifier significantly improved the classification ability of pre-miRNA sequences [21].

#### 2.1.3. Energy Characteristics

The real pre-miRNA sequences are generally more stable and show a lower minimum of free energy (MFE) than the randomly generated pre-miRNA. Therefore, energy characterization is often used to describe the structure pre-miRNA sequence as an aspect of feature extraction of the pre-miRNA sequence. To do this, the MFE value is obtained by using RNAfold to calculate the structure.

#### 2.1.4. Structural Diversity Characteristics

The potential for nucleotide pairing in the sequence is a significant characteristic that can also be used to describe the pre-miRNA sequence. This includes both traditional Watson-Crick nucleotide pairing (A–U pairing and C–G pairing) and also other forms of nucleotide pairing, such as the G–U pairing that can occur in the loop of RNA hairpin structures. We included possible G–U pairing in our description of base pairing.

To summarize, we extracted 98 features for the input of the neural network, including 64-dimensional *n*-gram frequency characteristics, 32-dimensional triple structure sequence characteristics, one-dimensional energy feature, and one-dimensional structural diversity characteristics.

### 2.2. Fixing the Number of Nodes in the Hidden Layer

In general, to select the number of nodes in the hidden layer in changing the BP neural network structure is difficult. Technically, a hidden layer could facilitate operation. However, too many hidden layers can reduce the operation rate.

Currently, no theoretical methods are available to fix the number of nodes in the hidden layer. However, the number generally depends on the empirical formula, as calculated in

(1) M=N+L+α,M=log2⁡ N,M=NL,

where *M* represents the neuron number of the hidden layers, *N* is the neuron number of the input layers, *L* is the neuron number of the output layers, and *α* is a constant between 1 and 10.

In this study, *N* = 98 and *L* = 1. Therefore, (1) can be used for any values between 11 and 20. A comprehensive analysis of the training results with different numbers of nodes in the hidden layer was performed with the error set to 0.0001. A total of 621 samples were used to train the network, and one sample was used to test the network. The results are shown in Table 1.

From the data shown in Table 1, the increased number of nodes in the hidden layer did not result in better convergence. Additionally, the increased number of nodes increased the network parameters and greatly increased the amount of calculation of the classifier. Thus, keeping 13 nodes in the hidden layers required relatively less training times and less error and still produced relatively good training effects.

### 2.3. Fixing the Number of Nodes in the Output Layer

**Figure 1:**
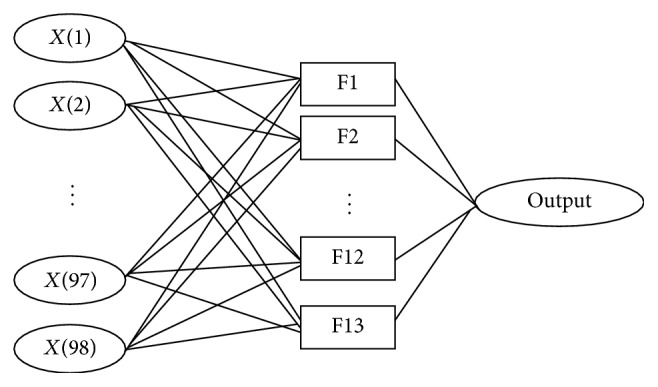
Topology structure of the BP neural network.

Two kinds of output exist, positive and negative, which are represented as 1 for a positive sample and 0 for a negative sample. The topology structure of this prediction method based on BP neural network is shown in Figure 1.

### 2.4. Selecting Training and Test Model Samples

The collection and organization of training samples are often limited by the objective conditions. Appropriate numbers of training samples are required to achieve sufficient precision. Therefore, it refers to the rule of experience:

(2) $$\begin{array}{c} P=\left(\;5\sim 10\;\right)\times P_{w}, \end{array}$$

where *P* represents the numbers of training samples and *P*_*w*_ is the total of network connection weight equal to the sum of nodes of the input and hidden layers. In this study, 2236 samples were used for training.

The data set used for the pre-miRNAs was downloaded from http://bioinf.sce.carleton.ca/SMIRP [35], and these data include negative and positive samples for* Arabidopsis lyrata*. The FASTA file was converted to ARFF file using a jar package written by Java converting the reference index to numerical form. We randomly selected real pre-miRNAs and pseudo pre-miRNAs to evaluate our algorithm.

### 2.5. Error Evaluation Steps Based on BP

The structure of the intelligent diagnosis model contains three layers of 98-13-1. First, we set the nodes of the input, output, and hidden layers as *N*, *M*, and *L*, respectively. Assuming the training sample set {*ξ* ^*p*^, *Y*} ⊂ *R* ^*N*^ × *R* ^*L*^, the weight matrix between the input and hidden layers can be written as *V* = (*v*_*mn*_)_*M*×*N*_, where *V*_*m*_ = (*v*_*m*1_, *v*_*m*1_,…,*v*_*m*1_)^*T*^ ∈ *R* ^*N*^ and *m* = 1,2,…, *M*. We assume the connection weight matrix between the hidden and output layers as *W* = (*w*_*lm*_)_*L*×*M*_, where *W*_*l*_ = (*v*_*l*1_, *v*_*l*1_,…,*v*_*lm*_)^*T*^ ∈ *R* ^*M*^, *l* = 1,2,…, *L*. Then, respectively, take *g* and *f* as the activation function of each node of the hidden and output layers. To simplify the derivation, we use the vector function *G*(*X*) for *X* = (*x*_1_, *x*_2_,…,*x*_*m*_)^*T*^ ∈ *R* ^*M*^, where *G*(*X*) = (*g*(*x*_1_), *g*(*x*_2_),…,*g*(*x*_*m*_))^*T*^ ∈ *R* ^*M*^. After input of the sample *ξ* ^*p*^ ∈ *R* ^*N*^, the actual output can be calculated by

(3) $$\begin{array}{c} \zeta_{l}^{p}=f\left(\;w_{l}\cdot G\left(\;V_{l}\xi^{p}\;\right)\;\right). \end{array}$$

The error function is defined in

(4) $$\begin{array}{c} E\left(\;W,V\;\right)=\frac{1}{2}\overset{P}{\underset{p=1}{\sum }}\;\overset{L}{\underset{l=1}{\sum }}{\left(\;O^{p}-f\left(\;w_{l}\cdot G\left(V_{l}\xi^{p}\right)\;\right)\;\right)}^{2}. \end{array}$$

Objectively, the target of BP training is to compute the *W* and *V* to minimize the solution of the error function *E*(*W*, *V*). With this, a combination of gradient descent, common, and simple derivatives was used. To simplify the derivation process, we derive

(5) $$\begin{array}{c} f_{pl}\left(\;x\;\right)=\frac{1}{2}{\left(\;O_{l}^{p}-f\left(\;x\;\right)\;\right)}^{2}. \end{array}$$

Then, the error function can be written as

(6) $$\begin{array}{c} E\left(\;W,V\;\right)=\overset{P}{\underset{p=1}{\sum }}\;\overset{L}{\underset{l=1}{\sum }}f_{pl}\left(\;w_{l}\cdot G\left(\;V_{m}\xi^{p}\;\right)\;\right). \end{array}$$

The corresponding gradient function of *W* and *V* can then be expressed as

(7) EwlW,V=∑p=1Pfpllwl·GVξpGVξp,EvmW,V=∑p=1P ∑l=1Lfpllwl·GVξpwlmg´vm·ξpξp.

For arbitrary initial values of *W*_0_ ∈ *R* ^*L*×*M*^ and *V*_0_ ∈ *R* ^*M*×*N*^, gradient descent rules to modify the weight of the BP learning algorithm are applied in

(8) Wln+1=Wln+ΔWln,ΔWln=−ηnEwlWn,Vn,Vmn+1=Vmn+ΔVmn,ΔVmn=−ηnEwmWn,Vn,

where *η*_*n*_ represents the learning efficiency. Δ*W*_*l*_^*n*^ is the partial derivative of the error function relative to *W*. Δ*V*_*m*_^*n*^ is the partial derivative of the error function relative to *V*.

### 2.6. Selection of Training Functions and Related Parameters

The above analysis allows fixing of the BP neural network structure.  Table 2 shows the chosen training functions and the relevant parameters.

**Figure 2:**
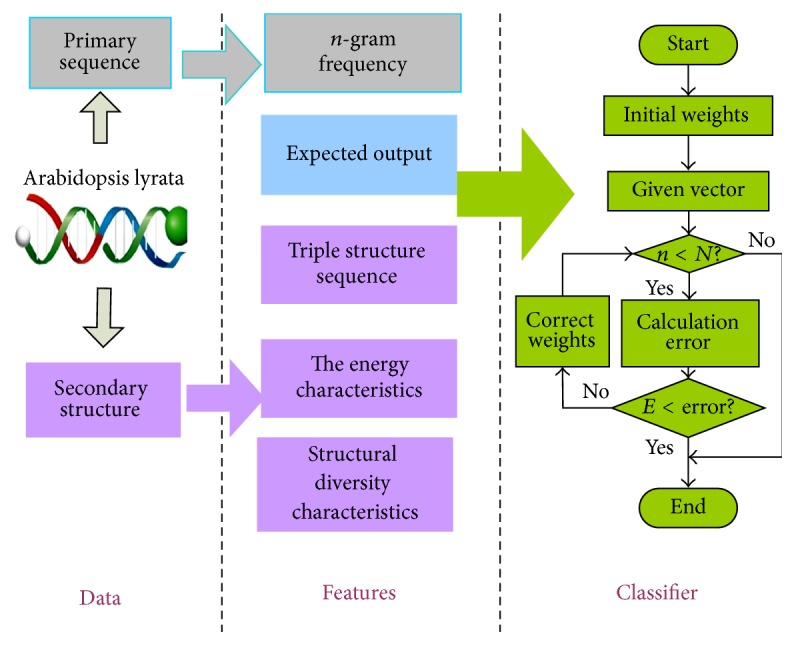
Process flow of model generation and training.

This condition allows establishment of a complete classifier based on BP neural network structure. The model generation and training are summarized in Figure 2.

### 2.7. Measurement

The use of pattern recognition and machine learning methods can be used as a two-way classification problem. Four kinds of prediction results are presented in Table 3.

The four kinds of prediction results are true positive (TP), the number of positive cases that were correctly predicted; false positive (FP), the number of positive cases represented by error prediction; true negative (TN), the number of counter negative examples that were correctly predicted; and false negative (FN), the number of negative cases represented by error prediction.

Many evaluation indicators can be used for the classification results. First, the accuracy rate (ACC) is the ratio of the correctly predicted cases for the entire data set. Precision and recall can also be used as evaluation indicators in tests of pattern recognition models. Precision is expressed as the ratio of the correctly predicted values for the entire positive data set and recall reflects the number correctly judged as positive examples in the positive example test set [36]. The above three indicators are expressed in

(9) ACC=TP+TNTP+FP+TN+FN,precision=TPTP+FP,recall=TPTP+FN.

Additionally, sensitivity and specificity parameters may be used to evaluate the function of the model. Sensitivity record (SE) is the same as the recall and specificity record (SP) calculated in accordance with

(10) $$\begin{array}{c} SP=\frac{TN}{TN+FP}. \end{array}$$

A challenge may be presented if the positive and negative test sets are unbalanced in the study of biological information. In most cases, the number of positive samples is far less than the number of negative samples. In a few cases, the number of positive samples may be much larger than the number of negative samples. We can easily obtain ACC-SP when the number of positive samples is greater than the negative samples. In this case, the classifier only reflects the classification effect of the negative samples and is unable to accurately express the prediction effect of the classifier on the entire test data set. To solve this problem, researchers typically use the geometric mean (Gm) as described in

(11) $$\begin{array}{c} Gm=\sqrt{SE\cdot SP}. \end{array}$$

Matthew's correlation coefficient (MCC) [16, 21, 37, 38] can provide more equitable response forecast ability when a large difference exists between the number of positive samples and the number of negative samples. MCC can be expressed as

(12) MCC=TP×TN−FP×FNTP+FP×TN+FN×TP+FN×TN+FP.

Currently, studies on miRNA commonly use one or more of these above evaluation indices. In this work, we estimate the overall performance of the classifier by analysis of ACC, SE, SP, Gm, and MCC.

## 3. Results and Discussion

### 3.1. Analysis of Feature Set Performance

To select a better feature set for classification, we needed to determine the effect of different feature subsets on the performance of the classifier. To do this, we used the BP neural network method with the same training set (553 positive samples and 1150 samples) to test different feature sets, with the results shown in Table 4.

From Table 4, we learn that the accuracy of the entire feature sets can be as high as 93.42%. This result indicates that our feature set is more effective for processing of a more complex structure or sequence diversity. Considering that the feature sets used here are not very large and each feature subset is highly independent, reducing the dimension of the feature vector is no longer needed.

### 3.2. Performances of BP

*V*-fold cross-validation with moderate computational complexity is widely used for model selection. The selection of *V* is important because *V* not only determines the number of samples but also determines the computational complexity. Usually, a value of *V* between 5 and 10 is selected based on experience. Statistical performance shows little improvement when *V* selection is greater than 10. Again, computational complexity must be considered; thus a value between 5 and 10 is best [32].

**Figure 3:**
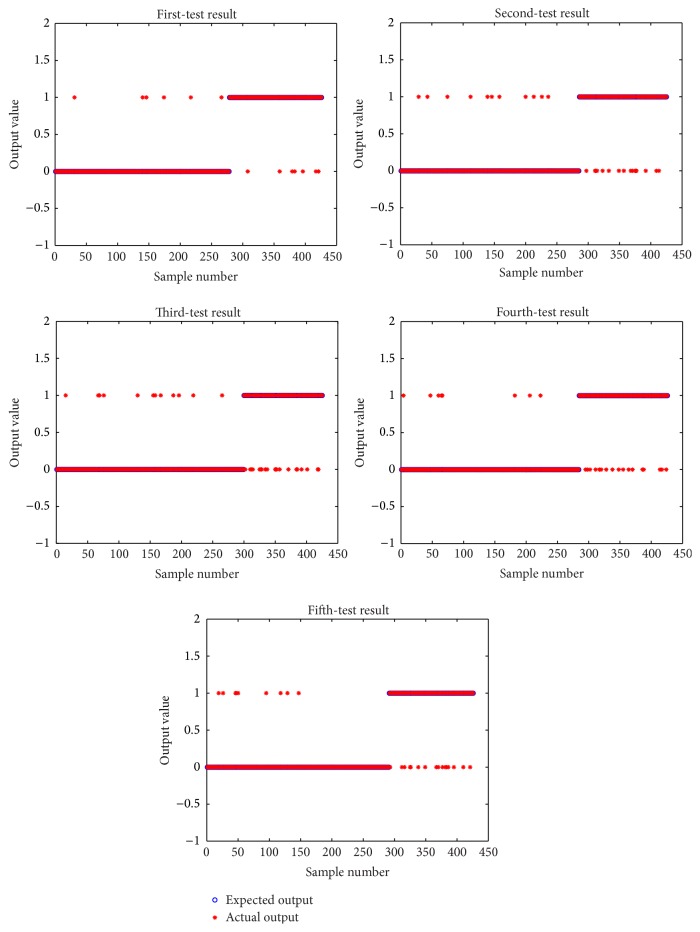
Different test results for varying sample quantities.

**Figure 4:**
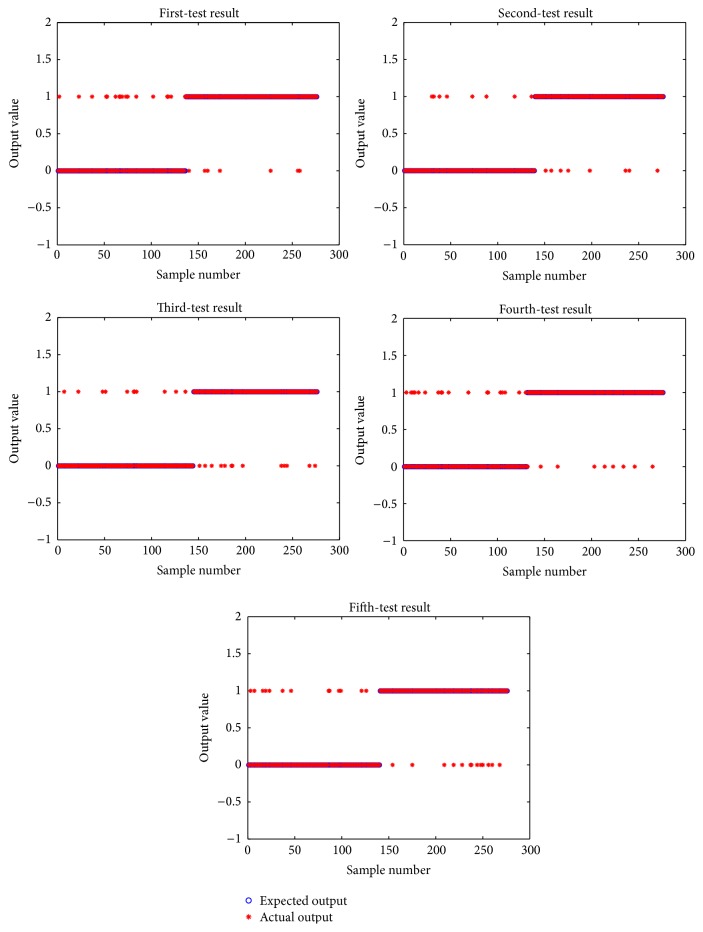
Different test results for same sample quantity.

We divided the samples into two cases for training and testing. In the first one, a large difference was observed between the number of positive and negative samples: 518 positive samples and 1078 negative samples as the training set and 166 positive samples and 366 negative samples for the test set. The second case included equal numbers of positive and negative samples: 552 positive samples and 552 negative samples as the training set and 138 positive samples and 138 negative samples for the test set. These training and testing were repeated five times. The testing performance is shown in Figures 3 and 4.

From comparison of the data in Figures 3 and 4, no significant difference was observed between the actual output and the expected output of each test. As described above, the evaluation of the reference index is shown in Table 5.

From the data presented in Table 5, the number of samples affects the accuracy and recall rate of the positive samples. In particular, the precision and recall rate of the negative samples decreased with the decrease in the number of negative samples in the training set. This result indicates that the more the samples in the training process, the better the classification effect of the classifier. At the same time, the precision and recall rate of the number of positive samples were affected. With the number of negative samples in the training set increased, the number of correct predictions increased by four and the number of error predictions was reduced by eight. This result shows that the precision and recall rate of the positive samples decreased with the increase in the number of the negative samples.

### 3.3. Comparison with Other Methods

**Figure 5:**
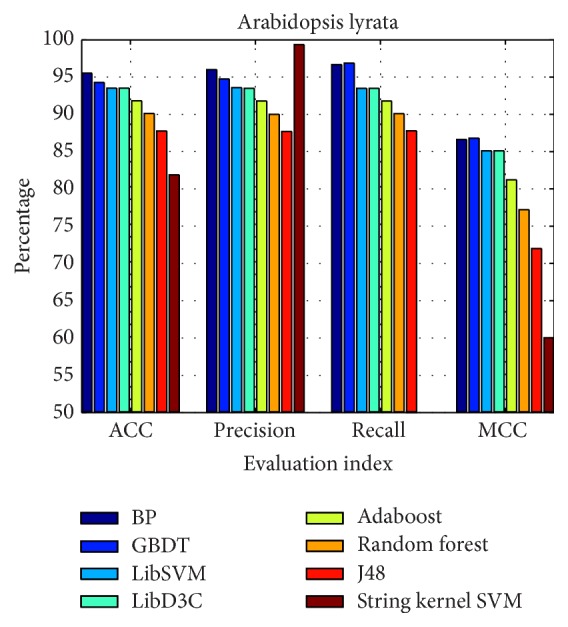
Comparison results of different models.

The performance of our method was compared with other methods: J48, random forest, LibD3C [39], Adaboost, string kernel SVM [40], LibSVM, and GBDT, which were classified on the same data set. The data set contains 691 real pre-miRNAs and 1437 pseudo pre-miRNAs. As shown in Table 6 and Figure 5, the results demonstrate that the total prediction accuracy of our method is 13.64% greater than the string kernel SVM model and nearly 2% greater than the LibD3C and LibSVM models. The overall performance of the models as measured by MCC was in the following order: GBDT (0.8682), BP (0.8662), LibSVM (0.8510), LibD3C (0.8510), Adaboost (0.8120), random forest (0.7720), J48 (0.7200), and string kernel SVM (0.6002).

Thus, we conclude that the BP method allows improved recognition accuracy.

### 3.4. Performance on Different Species

**Figure 6:**
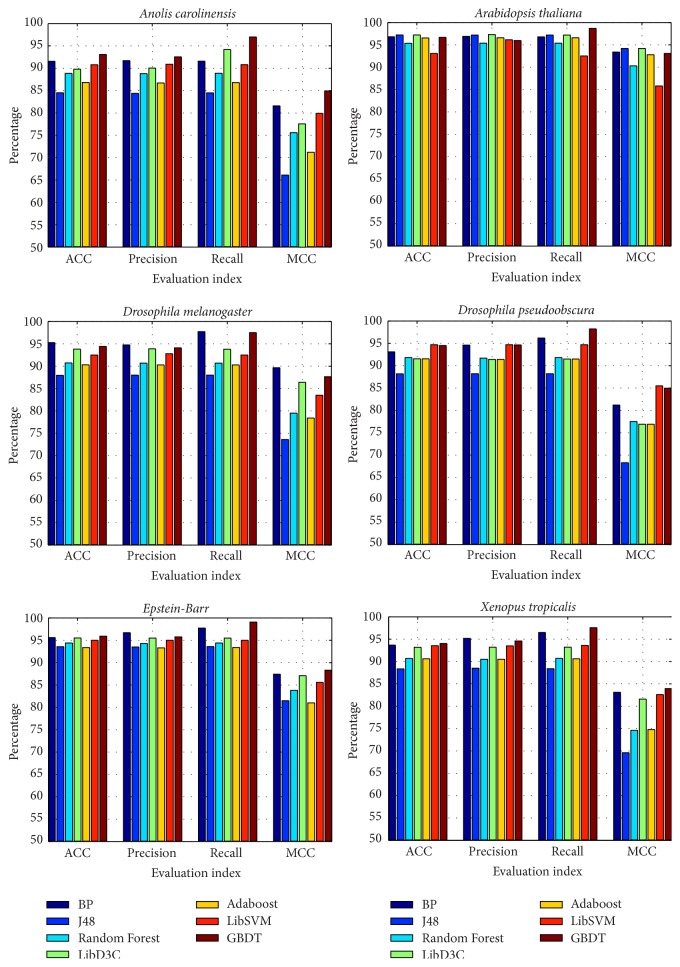
Test comparison results for six different species.

To demonstrate the validity and the universal applicability of the BP method, we analyzed six other species:* Anolis carolinensis*,* Arabidopsis thaliana*,* Drosophila melanogaster*,* Drosophila pseudoobscura*, Epstein-Barr virus, and* Xenopus tropicalis*. The results shown in Figure 6 indicate that the accuracy of the GBDT is better than BP method in some situations, but the BP method has been achieved fairly good results in terms of ACC, precision, recall, and MCC.

## 4. Conclusions

Identification of miRNAs is the first step toward understanding their biological characteristics. Many approaches have been proposed to predict pre-miRNAs in recent years. However, feature extraction in these methods can result in information redundancy. To overcome this drawback, a BP neural network algorithm together with optimal 98D features was employed for this analysis. We compare our method with the existing methods of J48, random forest, LibD3C, Adaboost, GBDT, string kernel SVM, and LibSVM, which were trained on the same training data set. The results demonstrate that the total prediction accuracy of our method is 13.17% greater than the string kernel SVM model and nearly 2% greater than LibD3C and LibSVM.

After the identification step, functional analysis is also important for miRNA research. If human miRNA and diseases were focused on, two main approaches would be employed to predict the relationship. The first one is the statistical comparison analysis for the miRNA or isomiR expression [41]. The second one is the network analysis and prediction for miRNA-disease relationship [42–45]. Several advanced machine learning, network techniques, and bioinspired models can be utilized on this problem, including random forest [46], semisupervised learning [47], HeteSim Scores [48], spiking neural P systems [49–52], and membrane computing_*ENREF_51* [53–57]. Functional analysis of the novel detected miRNAs would be our future works.

## Figures and Tables

**Table 1:** Corresponding training results with different numbers of nodes in the hidden layers.

Hidden layers	Training times	Training errors	Hidden layers	Training times	Training errors
11	43	9.57718*e* − 005	12	39	9.88418*e* − 005
13	17	9.42136*e* − 005	14	65	9.92537*e* − 005
15	34	9.88206*e* − 005	16	74	8.38658*e* − 005
17	48	7.82527*e* − 005	18	157	6.63468*e* − 005
19	7	9.46711*e* − 005	20	47	9.3627*e* − 005

**Table 2:** Basic parameters of the classifier based on BP neural network.

Setting items	The value set
The learning rate	0.1
Error bounds	0.0001
The number of iterations	1000
Transfer function of hidden layer nodes	Tansig
Transfer function of output nodes	Purelin
The training function	Trainlm

**Table 3:** Measurements for the classification problems.

Classification result		
Actual result	Forecast result	
	P	N
P	TP	FN
N	FP	TN

**Table 4:** Comparison of classification results based on different feature sets.

Features	SP (%)	SE (%)	Gm (%)	ACC (%)
B	67.89	68.25	68.07	68.00
C	92.74	76.42	84.19	88.03
A + B	91.79	90.41	91.10	91.31
A + C	94.03	80.85	87.19	89.67
B + C	96.12	85.21	90.50	92.49
A + B + C	96.33	86.51	91.29	93.42

*Notes*: A: energy feature and structural diversity; B: 32-dimensional triad structure characteristic; C: 64-dimensional *n*-gram frequency characteristics.

**Table 5:** Evaluation of the reference index.

	Training sample	Test sample	Output sample	Correct sample	Precision (%)	Recall (%)	Gm (%)
D							
Positive	553	138	128	124	96.0	90.0	93.43
Negative	1150	287	296	282	95.38	98.19	

E							
Positive	552	138	136	128	94.10	92.82	93.98
Negative	552	138	140	130	92.87	94.12	

Note: D: sample set has different numbers of positive and negative samples; E: the sample set has equal numbers of positive and negative samples; correct sample: the number of correct predictions.

**Table 6:** Comparison of the BP with alternative models.

	ACC	Precision	Recall	MCC
BP	95.53%	96.00%	96.67%	0.8662
GBDT	94.27%	94.76%	96.87%	0.8682
LibSVM	93.52%	93.60%	93.50%	0.8510
LibD3C	93.52%	93.50%	93.50%	0.8510
Adaboost	91.82%	91.80%	91.80%	0.8120
Random forest	90.13%	90.00%	90.10%	0.7720
J48	87.78%	87.70%	87.80%	0.7200
String kernel SVM	81.89%	99.37%	46.31%	0.6002

## References

[1] Bartel D. P. (2004). microRNAs: genomics, biogenesis, mechanism, and function. *Cell*.

[2] Wu D., Huang Y., Kang J. (2015). ncRDeathDB: a comprehensive bioinformatics resource for deciphering network organization of the ncRNA-mediated cell death system. *Autophagy*.

[3] Huang Y., Liu N., Wang J. P. (2012). Regulatory long non-coding RNA and its functions. *Journal of Physiology and Biochemistry*.

[4] Zhang X., Wu D., Chen L. (2014). RAID: a comprehensive resource for human RNA-associated (RNA–RNA/RNA–protein) interaction. *RNA*.

[5] Hua S., Yun W., Zhiqiang Z., Zou Q. (2014). A discussion of micrornas in cancers. *Current Bioinformatics*.

[6] Wang Q., Wei L., Guan X., Wu Y., Zou Q., Ji Z. (2014). Briefing in family characteristics of microRNAs and their applications in cancer research. *Biochimica et Biophysica Acta—Proteins and Proteomics*.

[7] Yang C., Wu D., Gao L. (2016). Competing endogenous RNA networks in human cancer: hypothesis, validation, and perspectives. *Oncotarget*.

[8] Zeng X., Zhang X., Zou Q. (2016). Integrative approaches for predicting microRNA function and prioritizing disease-related microRNA using biological interaction networks. *Briefings in Bioinformatics*.

[9] Zou Q., Li J., Hong Q. (2015). Prediction of microRNA-disease associations based on social network analysis methods. *BioMed Research International*.

[10] Wang Y., Chen L., Chen B. (2013). Mammalian ncRNA-disease repository: a global view of ncRNA-mediated disease network. *Cell Death and Disease*.

[11] Caligiuri M. A., Yu J., He S., Trott R. Activation of Innate Immunity by miRNA for Cancer and Infection Treatment.

[12] Zhou H., Ge X., Xue X. (2016). microRNAs regulation and its role as biomarkers in diseases. *Oncology and Translational Medicine*.

[13] Kelly P. S., Gallagher C., Clynes M., Barron N. (2015). Conserved microRNA function as a basis for Chinese hamster ovary cell engineering. *Biotechnology Letters*.

[14] Zou Q., Zeng J., Cao L., Ji R. (2016). A novel features ranking metric with application to scalable visual and bioinformatics data classification. *Neurocomputing*.

[15] Liu W.-X., Deng E.-Z., Chen W., Lin H. (2014). Identifying the subfamilies of voltage-gated potassium channels using feature selection technique. *International Journal of Molecular Sciences*.

[16] Tang H., Chen W., Lin H. (2016). Identification of immunoglobulins using Chou's pseudo amino acid composition with feature selection technique. *Molecular BioSystems*.

[17] Zhu P.-P., Li W.-C., Zhong Z.-J. (2015). Predicting the subcellular localization of mycobacterial proteins by incorporating the optimal tripeptides into the general form of pseudo amino acid composition. *Molecular BioSystems*.

[18] Ding H., Feng P.-M., Chen W., Lin H. (2014). Identification of bacteriophage virion proteins by the ANOVA feature selection and analysis. *Molecular BioSystems*.

[19] Ding H., Guo S.-H., Deng E.-Z. (2013). Prediction of Golgi-resident protein types by using feature selection technique. *Chemometrics and Intelligent Laboratory Systems*.

[20] Ding H., Lin H., Chen W. (2014). Prediction of protein structural classes based on feature selection technique. *Interdisciplinary Sciences: Computational Life Sciences*.

[21] Xue C., Li F., He T., Liu G.-P., Li Y., Zhang X. (2005). Classification of real and pseudo microRNA precursors using local structure-sequence features and support vector machine. *BMC Bioinformatics*.

[22] Jiang P., Wu H., Wang W., Ma W., Sun X., Lu Z. (2007). MiPred: classification of real and pseudo microRNA precursors using random forest prediction model with combined features. *Nucleic Acids Research*.

[23] Wei L., Liao M., Gao Y., Ji R., He Z., Zou Q. (2014). Improved and promising identification of human microRNAs by incorporating a high-quality negative set. *IEEE/ACM Transactions on Computational Biology and Bioinformatics*.

[24] Wang Y., Chen X., Jiang W. (2011). Predicting human microRNA precursors based on an optimized feature subset generated by GA-SVM. *Genomics*.

[25] Liu B., Fang L., Liu F., Wang X., Chen J., Chou K.-C. (2015). Identification of real microRNA precursors with a pseudo structure status composition approach. *PLoS ONE*.

[26] Liu B., Fang L., Chen J., Liu F., Wang X. (2015). MiRNA-dis: MicroRNA precursor identification based on distance structure status pairs. *Molecular BioSystems*.

[27] Wang X., Laurie J. D., Liu T., Wentz J., Liu X. S. (2011). Computational dissection of Arabidopsis smRNAome leads to discovery of novel microRNAs and short interfering RNAs associated with transcription start sites. *Genomics*.

[28] Zhang X., Tian Y., Cheng R., Jin Y. (2015). An efficient approach to nondominated sorting for evolutionary multiobjective optimization. *IEEE Transactions on Evolutionary Computation*.

[29] Zhang X., Tian Y., Jin Y. (2014). A knee point-driven evolutionary algorithm for many-objective optimization. *IEEE Transactions on Evolutionary Computation*.

[30] Yousef M., Nebozhyn M., Shatkay H., Kanterakis S., Showe L. C., Showe M. K. (2006). Combining multi-species genomic data for microRNA identification using a Naïve Bayes classifier. *Bioinformatics*.

[31] Arlot S., Celisse A. (2010). A survey of cross-validation procedures for model selection. *Statistics Surveys*.

[32] Bonnet E., Wuyts J., Rouzé P., Van de Peer Y. (2004). Evidence that microRNA precursors, unlike other non-coding RNAs, have lower folding free energies than random sequences. *Bioinformatics*.

[33] Liu H., Wong L. (2003). Data mining tools for biological sequences. *Journal of Bioinformatics and Computational Biology*.

[34] Liu B., Liu F., Wang X., Chen J., Fang L., Chou K. (2015). Pse-in-One: a web server for generating various modes of pseudo components of DNA, RNA, and protein sequences. *Nucleic Acids Research*.

[35] Peace R. J., Biggar K. K., Storey K. B., Green J. R. (2015). A framework for improving microRNA prediction in non-human genomes. *Nucleic Acids Research*.

[36] Yang S., Cai S., Zheng F. (2014). Representation of fluctuation features in pathological knee joint vibroarthrographic signals using kernel density modeling method. *Medical Engineering and Physics*.

[37] Wang R., Xu Y., Liu B. (2016). Recombination spot identification Based on gapped k-mers. *Scientific Reports*.

[38] Wu Y., Chen P., Luo X. (2016). Quantification of knee vibroarthrographic signal irregularity associated with patellofemoral joint cartilage pathology based on entropy and envelope amplitude measures. *Computer Methods and Programs in Biomedicine*.

[39] Lin C., Chen W., Qiu C., Wu Y., Krishnan S., Zou Q. (2014). LibD3C: ensemble classifiers with a clustering and dynamic selection strategy. *Neurocomputing*.

[40] Ghandi M., Lee D., Mohammad-Noori M., Beer M. A. (2014). Enhanced regulatory sequence prediction using gapped *k*-mer features. *PLoS Computational Biology*.

[41] Guo L., Yu J., Liang T., Zou Q. (2016). miR-isomiRExp: a web-server for the analysis of expression of miRNA at the miRNA/isomiR levels. *Scientific Reports*.

[42] Chen X., Yan C. C., Zhang X. (2016). WBSMDA: within and between score for MiRNA-disease association prediction. *Scientific Reports*.

[43] Chen X., Clarence Yan C., Zhang X. (2015). RBMMMDA: predicting multiple types of disease-microRNA associations. *Scientific Reports*.

[44] Chen X., Liu M.-X., Yan G.-Y. (2012). RWRMDA: predicting novel human microRNA-disease associations. *Molecular BioSystems*.

[45] Chen X. (2016). miREFRWR: a novel disease-related microRNA-environmental factor interactions prediction method. *Molecular BioSystems*.

[46] Liu Y., Zeng X., He Z., Zou Q. (2016). Inferring microRNA-disease associations by random walk on a heterogeneous network with multiple data sources. *IEEE/ACM Transactions on Computational Biology and Bioinformatics*.

[47] Chen X., Yan G.-Y. (2014). Semi-supervised learning for potential human microRNA-disease associations inference. *Scientific Reports*.

[48] Zeng X., Liao Y., Liu Y., Zou Q. (2016). Prediction and validation of disease genes using HeteSim Scores. *IEEE/ACM Transactions on Computational Biology and Bioinformatics*.

[49] Song T., Xu J., Pan L. (2015). On the universality and non-universality of spiking neural P systems with rules on synapses. *IEEE Transactions on NanoBioscience*.

[50] Zhang X., Zeng X., Luo B., Pan L. (2014). On some classes of sequential spiking neural P systems. *Neural Computation*.

[51] Song T., Pan L. (2016). Spiking neural P systems with request rules. *Neurocomputing*.

[52] Wang X., Song T., Gong F., Zheng P. (2016). On the computational power of spiking neural P systems with self-organization. *Scientific Reports*.

[53] Zeng X., Xu L., Liu X., Pan L. (2014). On languages generated by spiking neural P systems with weights. *Information Sciences*.

[54] Zhang X., Pan L., Păun A. (2015). On the universality of axon P systems. *IEEE Transactions on Neural Networks and Learning Systems*.

[55] Chen X., Pérez-Jiménez M. J., Valencia-Cabrera L., Wang B., Zeng X. (2016). Computing with viruses. *Theoretical Computer Science*.

[56] Wu T., Zhang Z., Păun G., Pan L. (2016). Cell-like spiking neural P systems. *Theoretical Computer Science*.

[57] Zhang X., Liu Y., Luo B., Pan L. (2014). Computational power of tissue P systems for generating control languages. *Information Sciences*.

